# Pathologic complete response of ductal carcinoma in situ to neoadjuvant systemic therapy in HER2-positive invasive breast cancer patients: a nationwide analysis

**DOI:** 10.1007/s10549-023-07012-z

**Published:** 2023-07-03

**Authors:** Roxanne A. W. Ploumen, Eva L. Claassens, Loes F. S. Kooreman, Kristien B. M. I. Keymeulen, Maartje A. C. E. van Kats, Suzanne Gommers, Sabine Siesling, Thiemo J. A. van Nijnatten, Marjolein L. Smidt

**Affiliations:** 1 Department of Surgery, Maastricht University Medical Centre+, Maastricht, The Netherlands; 2 GROW – School for Oncology and Reproduction, Maastricht University Medical Centre+, Maastricht, The Netherlands; 3 Department of Pathology, Maastricht University Medical Centre+, Maastricht, The Netherlands; 4 Department of Medical Oncology, Maastricht University Medical Centre+, Maastricht, The Netherlands; 5 Department of Radiology and Nuclear Medicine, Maastricht University Medical Centre+, Maastricht, The Netherlands; 6 Department of Health Technology and Services Research, Technical Medical Centre, University of Twente, Enschede, The Netherlands; 7 Department of Research and Development, Netherlands Comprehensive Cancer Organisation, Utrecht, The Netherlands

**Keywords:** Breast neoplasms, Ductal carcinoma in situ, Neoadjuvant therapy, Response

## Abstract

**Purpose:**

Ductal carcinoma in situ (DCIS) is present in more than half of HER2-positive invasive breast cancer (IBC). Recent studies show that DCIS accompanying HER2-positive IBC can be completely eradicated by neoadjuvant systemic therapy (NST). Our aim was to determine the percentage of pathologic complete response of the DCIS component in a nationwide cohort and to assess associated clinicopathologic variables. Furthermore, the impact on surgical treatment after NST was investigated.

**Methods:**

Women diagnosed with HER2-positive IBC, treated with NST and surgery, between 2010 and 2020, were selected from the Netherlands Cancer Registry. Pre-NST biopsy and postoperative pathology reports were obtained from the Dutch Nationwide Pathology Databank and assessed for the presence of DCIS. Clinicopathologic factors associated with DCIS response were assessed using logistic regression analyses.

**Results:**

A DCIS component was present in the pre-NST biopsy in 1403 (25.1%) of 5598 included patients. Pathologic complete response of the DCIS component was achieved in 730 patients (52.0%). Complete response of DCIS occurred more frequently in case of complete response of IBC (63.4% versus 33.8%, *p* < 0.001). ER-negative IBC (OR 1.79; 95%CI 1.33–2.42) and more recent years of diagnosis (2014–2016 OR 1.60; 95%CI 1.17–2.19, 2017–2019 OR 1.76; 95%CI 1.34–2.34) were associated with DCIS response. Mastectomy rates were higher in IBC+DCIS compared to IBC (53.6% versus 41.0%, *p* < 0.001).

**Conclusion:**

Pathologic complete response of DCIS occurred in 52.0% of HER2-positive IBC patients and was associated with ER-negative IBC and more recent years of diagnosis. Future studies should investigate imaging evaluation of DCIS response to improve surgical decision making.

**Supplementary Information:**

The online version contains supplementary material available at 10.1007/s10549-023-07012-z.

## Introduction

Neoadjuvant systemic therapy (NST) has gained an important role in the treatment of invasive breast cancer (IBC). Earlier, NST was reserved for locally advanced or inoperable breast cancer, while nowadays NST can be considered in early stage breast cancer [6]. The main goal of NST is to improve oncologic outcomes and additionally to reduce tumor extent in order to improve breast-conserving surgery rates [1, 9]. The response rate depends on the breast cancer subtype, with the highest rates of pathologic complete response (pCR) in HER2-positive or triple-negative IBC [8].

In case of HER2-positive IBC, a ductal carcinoma in situ (DCIS) component accompanies the invasive tumor in 57.4%–71.6% of patients [4, 10]. Some studies show that in IBC patients with a DCIS component, the pCR rate is lower, while others did not find an association between the presence of DCIS and pCR [11, 18, 22]. DCIS was previously considered insensitive to NST, due to its protective basal membrane, less dense micro-vasculature, and lower proliferative state as opposed to IBC [25]. Subsequently, IBC patients with a DCIS component were less likely to undergo breast-conserving surgery, both in case of primary surgery and after NST [10, 24].

Recently, a few studies have shown that the DCIS component accompanying HER2-positive IBC can respond to NST. Von Minckwitz et al. demonstrated that in their population including 59 HER2-positive IBC patients with a DCIS component, 30 (50.8%) showed a pCR of the DCIS component [22]. Groen et al. investigated 138 HER2-positive IBC patients with a DCIS component and showed a pCR of DCIS in 46% of patients after NST. In conclusion, current literature suggests response of the DCIS component in HER2-positive IBC, but these few articles only concerned small study populations.

Therefore, the aim of this study was to determine the rate of pCR of a DCIS component in HER2-positive IBC in a large cohort of patients by performing a nationwide analysis. In addition, the influence of clinicopathologic variables on the rate of pCR of the DCIS component and the impact of the DCIS component on surgical treatment were investigated.

## Materials and methods

### Data sources and study population

A database from the Netherlands Cancer Registry (NCR) was used for this nationwide retrospective study. Since 1989, trained registrars from the Netherlands Comprehensive Cancer Organization (IKNL) have been collecting data regarding patient, tumor, and treatment characteristics of all newly diagnosed cancer patients in the Netherlands. Upon request, the collected data can be used for research after approval by the privacy board of the IKNL.

Women aged 18 years or older, diagnosed with primary HER2-positive IBC, treated with neoadjuvant chemotherapy and targeted therapy followed by surgery between January 2010 and December 2019 in the Netherlands, were included from the NCR for the present study. This population was subsequently linked to PALGA, the Dutch Nationwide Pathology Databank [3]. In this way, all pre-NST biopsy and postoperative pathology reports were collected. Patients were excluded in case of missing pre-NST or postoperative pathology reports or when treatment differed from the Dutch guidelines at the year of diagnosis.

### Neoadjuvant systemic therapy and surgical procedure

NST regimens were based on the national guidelines in the year of diagnosis [12–14]. In HER2-positive IBC, NST is recommended in case of tumor size ≥ 5 mm or node-positive IBC. In general, NST consisted of Anthracyclines followed by Docetaxel or Paclitaxel, in combination with Trastuzumab. From 2016 onwards, patients with tumor size ≤ 2 cm received only Paclitaxel in combination with Trastuzumab for 12 weeks, based on the study by Tolaney et al.[19] Trastuzumab was in all patients continued after NST and surgery in the adjuvant setting for one year in total. Dual anti-HER2 blockade consisting of Trastuzumab with Pertuzumab was administered from 2017 onwards.

Surgical treatment after NST consisted of breast-conserving surgery or mastectomy and was at the discretion of the treating surgeon in consultation with the patient.

### Pathologic assessment of IBC, DCIS and response

Pathologic examination was performed locally according to the Dutch guideline [12–14]. The majority of the pathology laboratories use the Dutch Pathology Module (PALGA) for synoptic reporting, and standard work-up includes tumor subtyping, receptor status, and grading. Receptor status was evaluated for IBC, not for the DCIS component. ER status was determined using immunohistochemistry and considered positive if ≥ 10% of tumor cells stained positive. HER2 status was examined by immunohistochemistry or in situ hybridization, or in a combination, following ASCO CAP guidelines [23]. Tumor grade of IBC was classified according to the modified Bloom-Richardson guideline [2, 5]. In this study population of neoadjuvant treated patients, in general, the IBC grade of the postoperative specimen was recorded in the NCR. In case patients achieved pCR or when the grade in the biopsy was higher, the grade of the biopsy was recorded.

From PALGA, the presence of a DCIS component was collected from the pre-NST and postoperative pathology reports per patient. The grade of the DCIS component and the presence of comedonecrosis and/or calcifications were assessed in the pre-NST biopsy. In case the presence/absence of comedonecrosis and/or calcifications was not described, these variables were not classified as absent but as “missing value”.

Patients with a recorded DCIS component present in the pre-NST biopsy report were classified as IBC+DCIS and included in the analysis on complete response of DCIS. Complete response was defined as the absence of any DCIS in the postoperative specimen. Resection specimens below 30 g were embedded entirely for microscopic review. Larger specimens were sampled at at least 1 slide per cm of the expected tumor region.

### Study objectives

Primary endpoint was the percentage of pCR of DCIS in HER2-positive IBC patients with a DCIS component in the pre-NST biopsy. Secondary endpoints were association between complete response of IBC and complete response of DCIS, association of other clinicopathologic variables with complete response of DCIS, and impact of the presence of a DCIS component pre-NST on surgical treatment after NST.

### Statistical analysis

Statistical analyses were performed using the Statistical Package for the Social Sciences (SPSS, version 26, Armonk, New York). Descriptive statistics were used to summarize the study population. Complete response of the DCIS component was calculated as percentage of the patients with IBC+DCIS in the pre-NST biopsy. Pearson’s χ^2^ test was used to compare IBC response with DCIS response, and in this analysis, IBC response was defined as the absence of invasive breast cancer after NST (ypT0/is). Clinicopathologic variables associated with complete response of DCIS were determined by univariable logistic regression analyses. Subsequently, multivariable logistic regression analyses were performed to adjust for potential confounders. A complete case analysis was performed, in which patients with missing data were excluded from the univariable and multivariable analyses. Surgical treatment was compared between patients with IBC+DCIS and patients with pure IBC in the pre-NST biopsy using Pearson’s χ^2^ test. A *p* value ≤ 0.05 was considered statistically significant.

## Results

**Fig. 1:**
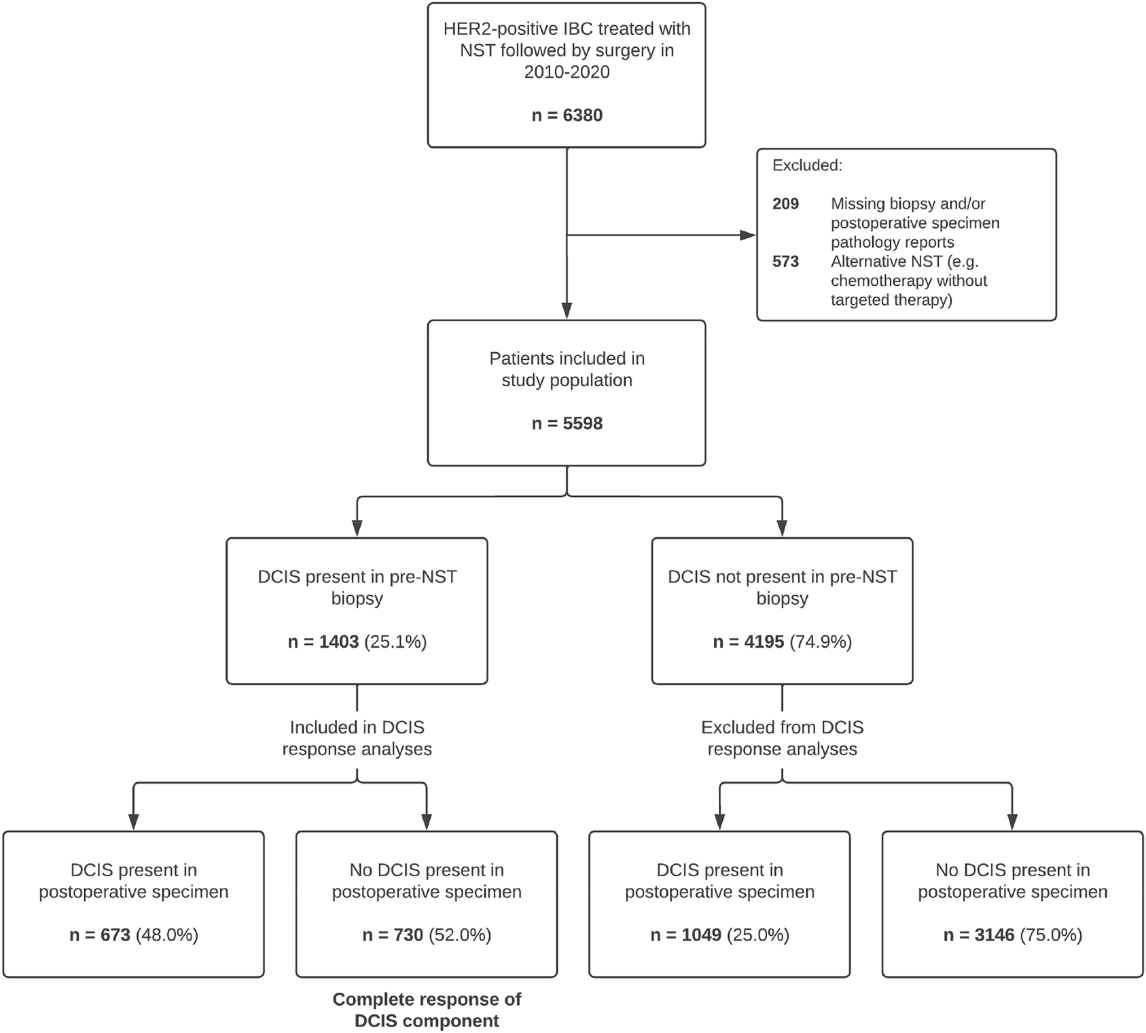
Flowchart of patient selection

In the period of 2010–2020, 6380 women with HER2-positive IBC received NST followed by surgical treatment in the Netherlands. After exclusion of ineligible patients (*n* = 782), 5598 patients were included in the study population (Fig. 1). Subsequently, pathology reports were assessed for the presence of DCIS, and 1403 patients (25.1%) showed a DCIS component in the pre-NST biopsy. These patients were included in the analysis on pathologic complete response of the DCIS component. The other 4195 patients (74.9) did not show a DCIS component in the pre-NST biopsy and were excluded from further analyses on DCIS response. An overview of the patient inclusion and classification based on pathology reports is shown in Fig. 1.

### Patient characteristics

Baseline characteristics of the 1403 patients with IBC+DCIS are shown in Table 1. Patients were most commonly diagnosed with cT2 tumor (50.7%), ER-positive (63.2%), and morphology of invasive carcinoma no special type (91.9%). IBC grade was most commonly grade 3 (47.7%), followed by grade 2 (46.1%). Patients with clinical tumor status Tis (*n* = 8) were included in the study population, since they had clinically node-positive disease and were treated with NST. Patients were classified as cTX (*n* = 18) when IBC was detected in pre-NST biopsy but cT status could not be determined on imaging.

**Table 1:** Baseline characteristics of patients with IBC with a DCIS component

Characteristics	IBC + DCIS (*n* = 1403) *N* (%)
Age at diagnosis in years, median [range]	48 [22–84]
Year of diagnosis	
2010–2013	259 (18.5)
2014–2016	410 (29.2)
2017–2019	734 (52.3)
Clinical tumor status	
T1	258 (18.4)
T2	711 (50.7)
T3	325 (23.2)
T4	81 (5.8)
Tis^a^	8 (0.6)
TX^a^	18 (1.3)
Missing	2
Clinical nodal status	
N0	618 (44.3)
N1	609 (43.7)
N2-3	167 (12.0)
Missing	9
IBC morphology	
Invasive carcinoma of no special type	1289 (91.9)
Lobular	5 (0.4)
Other	109 (7.7)
IBC grade	
1	68 (6.1)
2	512 (46.1)
3	530 (47.7)
Missing	293
IBC ER status	
ER-positive	886 (63.2)
ER-negative	515 (36.8)
Missing	2
DCIS grade	
1	41 (3.3)
2	428 (34.4)
3	774 (62.3)
Missing	160
Comedonecrosis	
Present	521 (76.8)
Absent	157 (23.2)
Missing	725
Calcifications	
Present	457 (61.7)
Absent	284 (38.3)
Missing	662

^a ^Diagnosed with cN + disease and treated with NST

Histopathologic characteristics of the DCIS component in the pre-NST biopsies are also shown in Table 1. Comedonecrosis and calcifications were present in 521 (76.8%) and 457 (61.7%) patients, respectively. The DCIS component was most often grade 3 (*n* = 774, 62.3%). DCIS grade and IBC grade were concordant in 61.8% of patients (616/997 patients, Supplementary Table 1).

### Association between clinicopathologic variables and the complete response of DCIS to NST

As presented in Fig. 1, 52.0% of the patients with a DCIS component in the pre-NST biopsy showed pCR of the DCIS component. The number of patients with complete response of IBC (ypT0/is) in comparison to complete response of the DCIS component is shown in Table 2. Complete response of the DCIS component occurred significantly more often in case of complete response IBC compared to patients with residual IBC (63.4% versus 34.1%, *p* < 0.001).

**Table 2:** Comparison of complete pathologic response of IBC and the DCIS component in the postoperative specimen after NST

No. (%) Total *N* = 1443	Complete response of DCIS	Residual DCIS	*p* value
	*N* = 730/1403 (52.0)	*N* = 673/1403 (48.0)	
Complete response of IBC	544/858 (63.4)	314/858 (36.6)	< 0.001^a^
*N* = 858/1403 (61.2)			
Residual IBC	186/545 (34.1)	359/545 (65.9)	
*N* = 545/1403 (38.8)			

^a ^statistically significant

The univariable and multivariable logistic regression analyses for clinicopathologic variables associated with complete response of DCIS are shown in Table 3. In univariable analyses, age at diagnosis above 50 (OR 1.41; 95% CI 1.14–1.75), year of diagnosis between 2014–2016 (OR 1.60, 95% CI 1.17–2.19) and between 2017–2019 (OR 1.76, 95% CI 1.34–2.34), clinical tumor status T3 (OR 0.59; 95% CI 0.43–0.82), and ER-negative IBC (OR 1.65; 95% CI 1.33–2.06) were significantly associated with complete DCIS response. Of the pathologic characteristics of the DCIS component, the presence of both comedonecrosis (OR 0.66; 95% CI 0.46–0.94) and calcifications (OR 0.58; 95% CI 0.43–0.78) were significantly associated with a complete response of DCIS to NST. DCIS grade and IBC grade were not associated with DCIS response.

**Table 3:** Association of clinicopathologic variables with complete response of DCIS to NST in univariable and multivariable regression analyses

	Complete pathologic response of DCIS N/total (%)	Univariable analysis			Multivariable analysis		
Clinicopathologic factors		OR	95% CI	*p* value	OR	95% CI	*p* value
Age at diagnosis (years)							
< 50	384/795 (48.3)	REF			REF		
≥ 50	346/608 (56.9)	1.41	1.14 – 1.75	0.001^a^	1.24	0.95 – 1.61	0.11
Year of diagnosis							
2010–2013	107/259 (41.3)	REF			REF		
2014–2016	217/410 (52.9)	1.60	1.17 – 2.19	0.003^a^	1.64	1.06 – 2.54	0.03^a^
2017–2019	406/734 (55.3)	1.76	1.32 – 2.34	< 0.001^a^	1.83	1.23 – 2.72	0.003^a^
Clinical tumor status							
T1	148/258 (57.4)	REF			REF		
T2	374/711 (52.6)	0.83	0.62 – 1.10	0.19	0.77	0.55 – 1.08	0.12
T3	144/325 (44.3)	0.59	0.43 – 0.82	0.002^a^	0.57	0.39 – 0.85	0.006^a^
T4	50/81 (61.7)	1.20	0.72 – 2.00	0.49	1.19	0.60 – 2.35	0.62
Tis^b^	4/8 (50.0)	0.74	0.18 – 3.04	0.68	1.37	0.12 – 15.46	0.80
TX^b^	9/18 (50.0)	0.74	0.29 – 1.93	0.54	1.05	0.22 – 4.94	0.95
IBC grade							
Grade 1	34/68 (50.0)	REF			REF		
Grade 2	269/512 (52.5)	1.11	0.67 – 1.84	0.69	0.78	0.43 – 1.41	0.41
Grade 3	287/530 (54.2)	1.18	0.71 – 1.96	0.52	0.82	0.44 – 1.51	0.52
ER status							
Positive	420/886 (47.4)	REF			REF		
Negative	308/515 (59.8)	1.65	1.33 – 2.06	< 0.001^a^	1.81	1.36 – 2.39	< 0.001^a^
DCIS grade							
1	15/41 (36.6)	REF			REF		
2	215/428 (50.2)	1.75	0.90 – 3.40	0.10	1.72	0.80 – 3.72	0.17
3	372/774 (48.1)	1.60	0.84 – 3.08	0.16	1.48	0.69 – 3.19	0.32
Comedonecrosis							
Absent	95/157 (60.5)	REF			^c^		
Present	261/521 (50.1)	0.66	0.46 – 0.94	0.02^a^			
Calcifications							
Absent	171/284 (60.2)	REF			^c^		
Present	213/457 (46.6)	0.58	0.43 – 0.78	< 0.001^a^			

^*a *^statistically significant

^b ^diagnosed with cN + disease and treated with NST

^c ^comedonecrosis and calcifications were not included in multivariable analysis due to high number of missing values

*OR* odds ratio, *REF* reference

In multivariable logistic regression analyses, year of diagnosis between 2014 and 2016 (OR 1.64; 95% CI 1.06–2.54) and 2017–2019 (OR 1.83; 95% CI 1.23–2.72, and ER-negative IBC (OR 1.81; 95% CI 1.36–2.39) were independently associated with higher odds for pCR of the DCIS component. Clinical tumor status T3 was independently associated with lower odds for pCR of the DCIS component (OR 0.57; 95% CI 0.39–0.85). The other abovementioned univariable clinicopathologic variables did not reach significance after multivariable logistic regression analysis. Comedonecrosis and calcifications were not included in the multivariable logistic regression analysis because of high numbers of missing data, resulting in too many patients being excluded from the analysis.

### Surgical treatment after NST

Surgical treatment differed significantly between patients with IBC + DCIS (*n* = 1403) and patients with pure IBC (*n* = 4195) in the pre-NST biopsy. Mastectomy was more often performed as primary surgical treatment in patients with IBC + DCIS (*n* = 742, 52.9%) compared to pure IBC (*n* = 1681, 40.1%) (*p* < 0.001). Postoperative pathology outcomes (ypT status) are shown in Supplementary Table 2, for IBC patients and for IBC+DCIS patients, according to primary surgery treatment (BCS versus mastectomy). Of the total of 2423 patients receiving primary mastectomy, 1027 (42.4%) showed complete response (ypT0) in the postoperative pathology specimen.

## Discussion

In current studies investigating response to NST in breast cancer treatment, only a few studies have been conducted on the pathologic response of DCIS to NST. To the best of our knowledge, this is the first nationwide analysis investigating a large cohort of HER2-positive IBC patients with a DCIS component, and a pCR of DCIS was found in 52.0% of 1403 patients. In addition, we demonstrated that pCR of the DCIS component was associated with complete response of IBC, ER negativity of IBC and a more recent year of breast cancer diagnosis within this study cohort. Patients with a DCIS component in the pre-NST biopsy were significantly more often treated with mastectomy after NST compared to patients without a DCIS component.

The rate of pCR of the DCIS component is consistent with the outcomes of previous, smaller studies. Groen et al. and von Minckwitz et al. investigated the response of a DCIS component in HER2-positive IBC patients and found a complete response in 46% and 51% of these patients, respectively [7, 22]. Sun et al. found a slightly lower response rate of 35.7% in their population of 129 HER2-postive IBC patients [18]. Goldberg et al. investigated the response of a DCIS component in IBC patients treated with NST and found a response rate of 33%. This lower response rate can be explained by the study population consisting of different IBC subtypes, including HER2-negative. In comparison to these previous studies, a significantly larger number of patients was included in our study. Therefore, this study may be seen as a confirmation of previous results.

In addition, the potential association between clinicopathologic variables and DCIS response was investigated. First, it was found that complete response of the DCIS component occurred significantly more often in case of complete response of IBC (63.4% versus 34.1%, *p* < 0.001). Previous studies show a high concordance in receptor status and grade between IBC and the accompanying DCIS component [15–17]. In our multivariable analysis, ER-negative IBC was found to be significantly associated with complete response of DCIS, which is also associated with higher rates of pCR of the invasive tumor in previous studies [8, 20]. Given that IBC and the accompanying DCIS are comparable in morphology, their response could be affected by the same factors [15–17]. In addition, year of diagnosis between 2014 and 2016 and 2017–2019 was significantly associated with complete response of DCIS. This could be explained by the continuous improvements in NST in the recent years, including dual anti-HER2 blockade from 2017 onwards. Unfortunately, our database did not include information on treatment with single or dual anti-HER2 blockade. Yet, Groen et al. did find an independent association of dual anti-HER2 blockade with DCIS response in their analysis of 138 HER2+IBC patients with a DCIS component [7].

This study has strengths and limitations. A strength is the nationwide database of the NCR combined with the Dutch Nationwide Pathology Databank that allowed for evaluation of DCIS response on a large scale, in comparison to previous smaller study populations. Second, various clinicopathologic variables were taken into consideration, which enabled evaluation of association between clinicopathologic variables and complete response of the DCIS component.

There are certain limitations worth mentioning. First, due to the retrospective nature of our database, some variables are missing because of insufficient reporting, in particular regarding the pathologic characteristics of the DCIS component. The presence of a DCIS component in the postoperative specimen is a mandatory field in the Dutch Pathology Module since 2009. Unfortunately, the presence of a DCIS component in the pre-NST biopsy is not a mandatory field in completing the module. However, it has been added as an optional field as of 2016 and is often additionally described in the report. Nevertheless, this could have led to an underreporting of the DCIS component pre-NST by the pathologist focusing on the invasive tumor. Moreover, previous research shows there is a high inter-observer variation between pathologists and laboratories in grading DCIS, and the receptor status of the DCIS component is not yet a standard determination [21]. Therefore, these pathologic characteristics of the DCIS component could not be investigated properly in relation to response.

Second, the pre-NST biopsy collection generates another limitation. Since DCIS can appear outside of the area of the invasive tumor, there may be a risk of missing the DCIS component, when targeting the invasive tumor during biopsy. The presence of a DCIS component can therefore be underestimated and this may affect the complete response rate. Moreover, the location and size of the DCIS component outside of the invasive tumor can influence the possibility to perform breast-conserving surgery, but this was not possible to investigate based on the pre-NST pathology reports. Lastly, the higher mastectomy rate in the patients with a DCIS component could not be further evaluated because our database did not include relevant clinical data (e.g., gene expression, extent of mammographic calcifications, the use of oncoplastic and reconstructive surgery, and patients’ preference regarding surgical treatment).

Further research into complete response of DCIS in HER2-positive IBC is important, because our study confirms the increased mastectomy rate found in previous studies in patients with IBC+DCIS versus patients without a DCIS component (52.9% versus 40.1%, *p* < 0.001) [10, 24]. In order to implement the potential response of the DCIS component in personalizing surgical treatment after NST, future studies should evaluate whether it is possible to monitor response of the DCIS component by imaging modalities. Moreover, a thorough investigation of pathologic characteristics of the DCIS component in relation to response could be useful to predict DCIS response before start of NST.

In conclusion, in this nationwide retrospective study, we demonstrated that pCR of DCIS to NST occurred in 52.0% of the HER2-positive IBC patients with a DCIS component in pre-NST biopsy. These findings are important to create awareness that the presence of a DCIS component in particular should not necessarily indicate the need for mastectomy. Future studies should investigate the evaluation of DCIS response by imaging and the possibility of increasing the chance of breast-conserving surgery. In addition, further assessment of specific pathologic characteristics of DCIS related to response could possibly predict the chance of pCR.

## Supplementary Information

Below is the link to the electronic supplementary material.

Supplementary file1 (DOCX 14 KB)

Supplementary file2 (DOC 38 KB)

## Data Availability

The data used for this retrospective study were obtained via The Netherlands Comprehensive Cancer Organization (IKNL) from the Netherlands Cancer Registry. This specific dataset is generated for the current study by the registration team of IKNL after permission by their privacy board. Therefore, restrictions apply to the availability of the data and this specific dataset is not publicly available. However, the data are available upon reasonable request and with permission of the IKNL.
