# Supplementary material for: Pathologic complete response of ductal carcinoma in situ to neoadjuvant systemic therapy in HER2-positive invasive breast cancer patients: a nationwide analysis
Source: Breast Cancer Res Treat. 2023 Jul 3;201(2):227–35. doi: 10.1007/s10549-023-07012-z (PMC10361905; doi:10.1007/s10549-023-07012-z)
Supplement: Supplementary file 1 — Supplementary file1 (DOCX 14 KB) [file 10549_2023_7012_MOESM1_ESM.docx]

**Supplementary Table 1** Comparison of tumor grade between IBC and DCIS

|  | | **DCIS grade** | | | **Total** |
| --- | --- | --- | --- | --- | --- |
|  |  | *1* | *2* | *3* |  |
| **IBC grade** | *1* | 9 | 27 | 18 | 54 |
|  | *2* | 15 | 228 | 212 | 455 |
|  | *3* | 8 | 101 | 379 | 488 |
| **Total** | | 32 | 356 | 609 | 997 |

In case of concordant tumor grade in DCIS and IBC, cells were marked in grey.
