# Supplementary material for: Pathologic complete response of ductal carcinoma in situ to neoadjuvant systemic therapy in HER2-positive invasive breast cancer patients: a nationwide analysis
Source: Breast Cancer Res Treat. 2023 Jul 3;201(2):227–35. doi: 10.1007/s10549-023-07012-z (PMC10361905; doi:10.1007/s10549-023-07012-z)
Supplement: Supplementary file 2 — Supplementary file2 (DOC 38 KB) [file 10549_2023_7012_MOESM2_ESM.doc]

**Supplementary Table 2** Postoperative pathology of IBC and IBC+DCIS per primary surgical treatment after NST

| **ypT status** | **Primary surgical treatment IBC** | | **Primary surgical treatment IBC+DCIS** | | Total (n (%)) |
| --- | --- | --- | --- | --- | --- |
|  | Breast conserving surgery (n (%)) | Mastectomy (n (%)) | Breast conserving surgery (n (%)) | Mastectomy (n (%)) |  |
| ypT0 | 1231 (49.0) | 755 (44.9) | 275 (41.6) | 272 (36.7) | 2533 (45.2) |
| ypTis | 245 (9.7) | 185 (11.0) | 122 (18.4) | 193 (26.0) | 745 (13.3) |
| ypT1-2 | 663 (26.4) | 428 (25.5) | 91 (13.8) | 77 (10.4) | 1259 (22.5) |
| ypT1-2 + DCIS | 360 (14.3) | 227 (13.5) | 169 (25.6) | 174 (23.4) | 930 (16.6) |
| ypT3-4 | 11 (0.4) | 58 (3.5) | 1 (0.2) | 14 (1.9) | 84 (1.5) |
| ypT3-4 + DCIS | 4 (0.2) | 28 (1.6) | 3 (0.4) | 12 (1.6) | 47 (0.9) |
| Total | 2514 | 1681 | 661 | 742 | 5598 |
